# Knowledge Creation in Nursing Education

**DOI:** 10.5539/gjhs.v7n2p44

**Published:** 2014-09-28

**Authors:** Zahra Marzieh Hassanian, Mohammad Reza Ahanchian, Suleiman Ahmadi, Rezvan Hossein Gholizadeh, Hossein Karimi-Moonaghi

**Affiliations:** 1 Faculty of Nursing and Midwifery, Mashhad University of Medical Sciences, Mashhad, Iran; 2 Faculty of Education and Psychology, Ferdowsi University of Mashhad, Mashhad, Iran; 3 Faculty of Medicine Education, ShahidBeheshti University of Medical Sciences, Tehran, Iran; 4 Faculty of Medicine, Mashhad University of Medical Sciences, Mashhad, Iran

**Keywords:** knowledge creation, knowledge management, nursing knowledge, qualitative study

## Abstract

In today’s society, knowledge is recognized as a valuable social asset and the educational system is in search of a new strategy that allows them to construct their knowledge and experience. The purpose of this study was to explore the process of knowledge creation in nursing education. In the present study, the grounded theory approach was used. This method provides a comprehensive approach to collecting, organizing, and analyzing data. Data were obtained through 17 semi-structured interviews with nursing faculties and nursing students. Purposeful and theoretical sampling was conducted. Based on the method of Strauss and Corbin, the data were analyzed using fragmented, deep, and constant-comparative methods. The main categories included striving for growth and reduction of ambiguity, use of knowledge resources, dynamism of mind and social factors, converting knowledge, and creating knowledge. Knowledge was converted through mind processes, individual and group reflection, praxis and research, and resulted in the creation of nursing knowledge. Discrete nursing knowledge is gained through disconformity research in order to gain more individual advantages. The consequence of this analysis was gaining new knowledge. Knowledge management must be included in the mission and strategic planning of nursing education, and it should be planned through operational planning in order to create applicable knowledge.

## 1. Introduction

In educational and learning communities, knowledge is an asset with massive strategic value (Sallán, deÁlava, Barrera-Corominas, & Rodriguez Gomez, 2012). The body of knowledge of the nursing discipline contains philosophies, research, theories, ethics, and aesthetics (Smith & McCarthy, 2010). Based on Carper’s definition, there are four patterns of knowing, which include empirical, ethical, personal, and aesthetic knowing (Carper, 2004).

It is a fact that all universities are societies based on knowledge. Knowledge is the primary source used by educators, and the essential product used by students (Earl, 2001). Universities react to changes in society and manage their processes through the creation of knowledge (Rowley, 2000). Knowledge is a guiding principle in competitive advantages of organizations (Matusik & Hill, 1998; Zack, 1999). New competitive endeavors are moving from natural resources to knowledge capital (Tsai & Li, 2007). Organizational competency to create knowledge is the most important source of organizations’ sustainable competitive advantage (Parent, Gallupe, Salisbury, & Handelman, 2000). The creation of knowledge in the range of an organization’s activities is represented as an immaterial resource that can be discussed as a competitive advantage (Shinyashiki, Trevizan, & Mendes, 2003).

Past studies have introduced the vital role of knowledge creation in the success of organizations (Kogut, 2003; Chin & Kramer, 2005). Through knowledge creation, organizations can better connect knowledge to their new and distinctive methods and offer new values to clients (Lee & Choi, 2003). Knowledge creation is a constant process, through which individuals share tacit and explicit knowledge (Bloodgood & Salisbury, 2001). Knowledge is created in society, but adjusted within educational organizations (Amin & Cohendet, 2004).

The main goals of universities are teaching and learning, research, and providing services to the general public, and their mission is the creation, maintenance, and transference of knowledge (Eardley & Uden, 2011). Generally, the construction of knowledge takes place in academia through education, research, debating, and clinical learning (Bradley, 1996). The nursing discipline constructs knowledge based on the explanatory theory. This theory attempts to explain why things exist as they do in the world. Concepts that create the theory are related by prepositions that clarify the association (McKenna & Slevin, 2008). All theories that describe, explain, and predict that which exists in the world need concepts. Comprehensive concept development is vital in theory development. One method of concept development is concept synthesis that is required for theory building (Walker & Avant, 2005).

Educational knowledge entails tacit and explicit components (Nonaka & Takeuchi, 1995). Successful knowledge management depends on processes that improve personal and organizational capability, motivations, and opportunities for learning and knowledge acquisition, and being carried out in a manner that delivers positive outcome (Laal, 2011).

At present, nursing faculties in Iran offer nursing programs in BSc, MSc, and PhD degrees. Knowledge, in all forms of teaching and learning, is progressing in universities. It is essential that educators provide students with up-to-date knowledge to apply in the clinical environment. The development of knowledge in the faculties of nursing is due to knowledge creation. Thus, the question of how knowledge creation occurs in nursing faculties is relevant here.

Knowledge can be seen as a strategic resource to be attained, employed, and used, and thus, produce competitive advantage (Bergeron, 2003). In nursing education, knowledge creation is an important function of educators which occurs in different situations. Therefore, due to the importance of knowledge management in nursing education and as there is no comprehensive study on this area, it is important to explore the process of knowledge creation in nursing education. This article is part of a broader study on knowledge management which adopted grounded theory (GT) approach. This paper presents the process of nursing knowledge creation in Iranian nursing and midwifery faculties.

## 2. Methods

This study adopted the GT method (1998 version) that was presented by Strauss and Corbin. GT offers a systematic methodology for collecting, organizing, and analyzing data from different sources (Strauss & Corbin, 1990). GT is commonly used where there are few research findings in the subject area (KarimiMoonaghi, Dabbaghi, Oskouie, Vehvilainen-Julkunen, & Binaghi, 2010).

GT has been modified by researchers to fit with a diversity of philosophical positions such as constructivism and postmodernism (Annells, 1996). A constructivist approach to GT emphasizes the interaction between researcher and participants and gathering of several viewpoints. GT is a research methodology with roots in interpretive and symbolic interactions and affects knowledge construction (Mills, Bonner, & Francis, 2006). In this paper, a paradigm model is used as a guide to the presentation of the findings.

We used GT research design, as a general method of theoretical sampling, simultaneously with data production and analysis, category saturation, and constant comparative, open, axial, and theoretical coding (Pandit, 1996); (Strauss & Corbin, 1998).

### 2.1 Setting

This study was carried out in Mashhad Faculty of Nursing and Midwifery, Iran, where nursing educators and students in bachelor, master, and doctoral degrees of nursing were teaching and learning nursing. In this study, the major source of data collection was interviewing, and observation was used for completing the data collection. In addition, the interviewer (one of the researchers) was familiar to the research field.

### 2.2 Sampling

Purposive sampling method was used in the first stage, and was matched with theoretical sampling to find the codes and categories.

It is a known fact that nursing faculty and students who are involved in nursing education serve as the key informants regarding the process of knowledge creation in nursing education. To confirm variety in sampling, we engaged participants from every department of the nursing faculty, including educators and BSc, MSc, and PhD nursing students. All nursing educators and students were eligible to participate in the study except those who showed a disinterest in participating in the interviews. In this study all the cases requested to participate accepted the offer; none refused.

Educators were interviewed more than students, because they make greater contribution to the knowledge creation process.

In this study, the study population consisted of 15 individuals and 17 interviews were conducted; two of these interviews were repeated.

### 2.3 Data Collection

The main strategy for data collection was one-to-one in-depth interview with the participant. Where necessary, observation in classrooms and clinical settings during educating and learning time period was also used. This research required mutual understanding and cooperation between the researcher and the participants, since the interviews and observations were mutual, contextual, and value bounded (Lincoln & Guba, 1986). The interview guide was developed with the guidance of two expert supervisors, who were associate professors in nursing and educational management. Interviews were prearranged, and took place in a quiet room (No. 16) in the Mashhad Nursing and Midwifery Faculty. In total, 17 interviews were conducted lasting from 45 to 125 minutes.

All the interviews were audio-taped, transcribed verbatim, and analyzed successively by the first author. Reflective notes were made immediately and later after the interviews.

To confirm the lack of unclear data, the written interview along with the open codes were emailed to some of the interviewees; the result was the improvement of the validity of the study.

Participants were observed in classrooms and clinical settings where nursing knowledge creation occurs. The aim of observation was to gather data from different sources to complete and confirm the interview results.

### 2.4 Data Coding and Analysis

As the concepts emerged from the documents, analysis and constant comparative method were used simultaneously for analyzing the data (Strauss & Corbin, 1990). Every interview prepared guidance for the next one.

Open and axial coding was used along with reflection and the coding paradigm model served as guidance to assessing connected categories and concepts during data analysis. In open coding, data were broken down into discrete parts, closely examined, and compared for similarities and differences (Strauss & Corbin, 1998).

The purpose of axial coding is to begin the process of reassembling data fractured during open coding. In axial coding, categories are related to their subcategories along the lines of their properties and dimensions to form more precise and complete explanations about phenomena (Pandit, 1996). The relationship between the categories was studied which explains the process of knowledge creation in nursing education.

### 2.5 Data Trustworthiness

To prove credibility, researchers (two of the researchers) had prolonged engagement in nursing educational setting, interacted with participants, and allocated enough time to interviewing the participants.

The participants with the maximum difference were selected, which confirmed the conformability and credibility of the data (Streubert & Carpenter, 2003).

The interviews and coding were reviewed by two faculty supervisors, who were associate professors in nursing and educational management. In addition, they had experience in qualitative researches and knowledge management. Data integration was performed on both educators and students, and on observations and interviews. Credibility was strengthened through reflective notes and examination by members and colleagues. Furthermore, the results and interpretations of this study were reviewed by the two previously mentioned supervisors.

Dependency was established through external reviewing and use of additional comments of PhD students. Coding, the results of data analysis, and interpretations were carefully explained and reviewed by supervisors.

In addition, the data were confirmed through extracted codes which were approved by the participants.

### 2.6 Ethical Considerations

Initially, the study was approved by the Deputy of Research of the Faculty of Nursing and Midwifery, and then by the Medical Research Ethical Committee of the Mashhad University of Medical Sciences, Iran (16.04.2012, No. 910664). The Director of Education Office and the Director of Postgraduate Students approved the interviewing of participants according to a formal letter of introduction (serving as the legal document) from the Deputy of Research of the Faculty of Nursing and Midwifery and the Medical Research Ethical Committee of the Mashhad University of Medical Sciences.

The participants were enrolled in the study voluntarily, after signing informed consents, and they could withdraw whenever they desired. Moreover, their identities were not disclosed and results were published confidential.

## 3. Results

The findings presented in this section comprise the main categories recognized through data analysis. The participants’ individual characteristics are presented in Tables 1 and 2.

**Table 1:** Individual characteristics of the educators

Degree	Field	Sex		Age range (years)	Duration of educational experiences(years)	Duration of clinical experiences

		Men	Women			
**PhD**	Medical surgical nursing	2	1	34-48	7-17	6 months
	Medical surgical nursing	2	2	39-58	10-30	8 years (1 case)
**MSc**	Pediatric nursing	-	1	54	25	10 months
	Nursing management	1	-	49	25	5 years

**Table 2:** Individual characteristics of the students

Degree	Sex		Age range (years)	Semester	Duration of educational experiences	Duration of clinical experiences

	Women	Men				
**PhD**	1	1	36-50	2-4	10-17 years	1-6 years
**MSc**	2	1	27-28	1-5	-	6-8 months
**BSc**	1	-	22	8	-	-

Main categories emerged from the data presented in Table 3.

**Table 3:** Inter-relationship between the main category, generic categories, and subcategories

Subcategories	Generic categories	Main category
**Creating discrete knowledge through disconformity strive**	Striving for growth and reduction of ambiguity	Educators’ responsibility, tendency to growth, finding personal answers, personal growth, requirement of application of knowledge, professionalism.
	Application of knowledge origin Failure in application of resources	Use of library resource, uses of cyberspace, acquiring information from human resources, learning from clinical environment cultural failure, facilities failure, lack of time,
	Dynamism of the mind and social factors	Dynamism of minds, positive comorbidity teachers, positive comorbidity students, positive comorbidity colleagues
	Converting knowledge	Processing of mind and creation of knowledge, reflection, praxis, research

### 3.1 Striving for Growth and Reduction Ambiguity

Analysis of the data showed that participants had positive comorbidity in respect to constructing knowledge. Creation of knowledge had reduced ambiguity and developed individual excellence. Participants used factors such as creating and using opportunities, becoming wise, personal growth, and requirement of application of knowledge. One student described her experience as:

“Apparently, in the form of duty to do as the professor asks. To reach the goal, there are some sub-products, which, sometimes, become more important. For Example, you want to promote yourself; to know is a way to grow. This is the main purpose, which may not be clear most of the time.” (Student Participant No. 13)

### 3.2 Application of Knowledge Resources and Failure in Resources

#### 3.2.1 Applying Knowledge Resources

Based on participants’ experiences, use of knowledge resources had four sub-domains including use of library resources, use of cyberspace, acquiring knowledge from human resources, and learning from clinical environment.

1) Use of library resources: The participants increase their knowledge via studying nursing and medical books and articles, and other related subjects.
2) Use of cyberspace: The participants increase their knowledge using virtual space. Some participants used internet more than books, due to easy access and availability of more updated articles.
3) Acquiring information from human resources: Faculty members’ role as a source of knowledge was more highlighted in undergraduate level. In post-graduated level, faculty members’ role was that of a facilitator.
4) Learning from clinical environment: The participants gained experiences from clinical environment; the content was better understood by the students.

#### 3.2.2 Failure in Resources

Based on experiences of the participants, there were deficiencies in recourses for knowledge creation including cultural failure, facility failure, and lack of time.

1). Cultural failure: In this faculty, knowledge was created in a non-systematic manner and culture of knowledge management had not been created yet. Using knowledge management could help solve the problems.
2). Facility failure: There were some obstacles to preparing facilities for the construction of knowledge including low-speed internet, lack of updated books, lack of time for use of books, and in some fields, lack of good books.
3). Lack of time: Obstacles of knowledge construction were high workload and lack of available time.

### 3.3 Dynamism of the Mind and Social Factors

There were some dynamic factors that supported knowledge creation including dynamism of the mind and social factors.

1) Dynamism of mind: The dynamicity of participants’ mind played a contextual role in knowledge construction. Students need to have a creative and curious mind for building knowledge. When faced with a question, they discussed it with other students or their educators in or out of the class, and prepared themselves for next educational steps.
2) Dynamism of social factors: The dynamism of social factors consisted of three sub-domains of educators’ positive comorbidity, students’ positive comorbidity, and colleagues’ positive comorbidity.

- Educators’ positive comorbidity: Educators’ comments must stimulate growth in students. Students need educators, who give comments and have excellence thoughts and ideas, to construct their knowledge.
- Students’ positive comorbidity: In order to construct knowledge, students required classmates who had ideas and committed themselves to not speaking without thinking.
- Colleagues’ positive comorbidity: Some participants believed that holding meetings with their colleagues could be useful in creating knowledge. They had reflections, discussions, and deliberations that played an important role in the construction of knowledge.

Some participants believed that a suitable environment for contemplation and reflection, experience, wisdom, and cognitive ability were required for construction of knowledge.

### 3.4 Converting Knowledge

Participants used converting knowledge processing strategies to create knowledge. This strategy was conducted through mind processing, reflection, praxis, and research.

#### 3.4.1 Processing of Mind and Creation of Knowledge

During studying, participants kept contents in mind and repeated them or took notes of some contents for later use; this played an important role in creation of knowledge. Based on knowledge processing, creation of new knowledge in the mind occurs in a different way from that which previously occurred, it is more innovative.

Participants sometimes combined different methods of teaching and generated a new method which was considered as an innovation method.

#### 3.4.2 Reflection

Participants reflected on topics in two forms; personal reflection and contemplation, and group reflection and discussion. They often reflected and clarified the issues, which they experienced. In this case, one of the participants claimed that:

*“Sometimes I don’t get answers by individual reflection, so I talk with another person. Often, we have discussed the issue and found a more effective answer than we would have found alone; it is like a miracle. When I start to explain, unclear points actually become clear which I find really interesting” (Student participant No. 13)*.

#### 3.4.3 Praxis

In clinical environment, an important strategy to create knowledge was praxis. In this environment, participants performed activities to provide care to patients; and during and after providing care they reflected on patient care, and thus, new knowledge was created. One participant indicated that:

“When I am in the clinic, the environment is prepared for learning; patients, patient charts, clinical nurses, and physicians. It depends on your aim and your perspective to the clinical environment. When you go to the clinical environment to learn, your perspective differs from that of other times. When you enter the clinic with the perspective to learn, the whole environment is an educational environment and you can learn many subjects through reflection.” (Faculty participant No. 4)

#### 3.4.4 Research

One of the most important strategies to create knowledge in nursing education was research. Researches were carried out by educators and students (dissertations).

3.4.3.1 Researches conducted by educators: Both quantitative and qualitative research approaches were used by educators. Participants conducted their research in the nursing field. Some participants believed that the education system must provide conditions for improving research. It was important for some participants to conduct clinical and applicable researches. However, usually, due to some problems and long duration, most researches were conducted using a questionnaire and were not applicable research. One of the participants said:

“Most published articles are not practical. In order for researches to be practical in clinical environments, they must be conducted in clinical environments and at the bedside of the patient for a long period of time; so that we can trust it.” (Faculty participant No. 2)

However, recently, some action researches were conducted with a practical approach. One participant claimed:

“Action research creates applicable knowledge, if our research is accompanied by modifications, our clinical environment will be modified.” (Faculty participant No 12)

Occasionally, some educators requested postgraduate students to do research as class assignment in limited time. Students did not have interest in those subjects.

3.4.3.2 Dissertations: After the students chose the field and topic of the research, and their supervisor, they were guided by their supervisor to conduct the research.

Postgraduate students, mainly Master sciences student, use quantative research approach for their thesis. PhD students used qualitative research such as phenomenology, grounded theory, operational research, and mixed methods research. In qualitative researches, concepts are developed. In this regard, one of the participants mentioned: 

“I used grounded theory approach for my thesis. One chapter of my thesis offers a definition of organ donation nursing, which had not been defined previously.” (Faculty participant No 4)

Sometimes, examiners, which were invited to the session for dissertation approval, had not enough preparation; therefore, students confronted some problems. In this regard, one of participants stated that:

“*Examiner must have a little information about topic; he must study before meeting. One of the examiners quest of my framework base, I answer so much, but he didn’t accept. I took the least score of him. Later I showed him the documentations; he said that oh, I didn’t know”. (Student participant No. 11)*

### 3.5 Gaining New Knowledge

The outcome of the knowledge creation process was gaining new knowledge. The knowledge was the result of research, experience, praxis, thought, and discussion. Achieving actuality through reflection and contemplation, and changing previous concepts in the mind were results of research and knowledge creation. Creating a conceptual model and defining a new role for a nurse could be forms of achieving new knowledge.

Other consequences of knowledge creation were changes in procedures, providing solutions to problems, and changes in the activities and physical dimensions of the ward that is, occasionally, used individually or in some situations.

Some researches had imitative titles and did not solve problems of the profession; they were undervalued. The purpose of these researchers was only to publish their paper for personal benefits, regardless of the common professional problems. In this case, one of the participants said:

“Researches being conducted are not in line with the problems of our society. The first priority right now is earning points and self-promotion; and even if it is in line with the needs of the profession, the results still can’t be applied; it can be publish but the results cannot be used anywhere.”(Faculty participant No.2)

**Figure 1:**
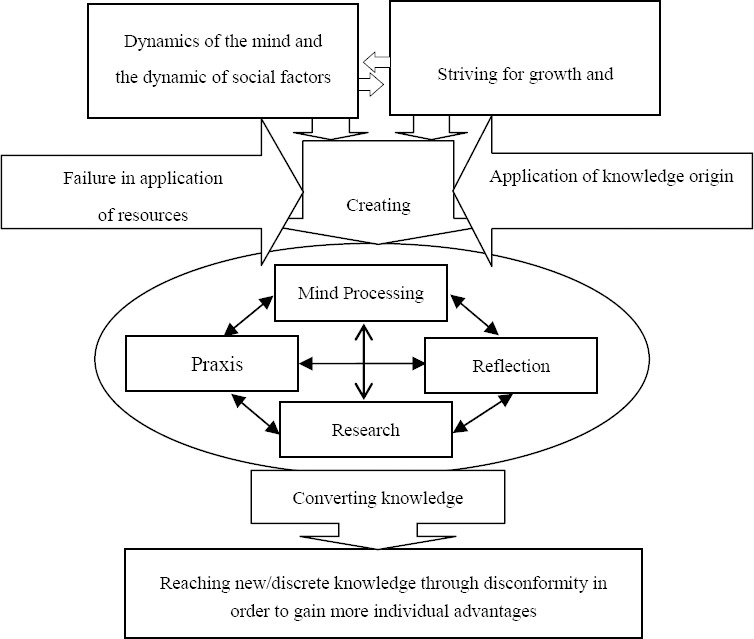
Interrelationship between the subcategories

Experiences of the participants indicated that most of the research findings were not applied. Excess of discrete nursing knowledge, and ongoing improvements were consequences of researching. The main finding in this study was that reaching new/discrete nursing knowledge occurs through disconformity in order to gain more individual advantages (Figure 1).

## 4. Discussion

Nursing and midwifery faculties are introduced as knowledge creating organizations. This comprehensive study demonstrated how nursing faculty and nursing students participate in the process of knowledge creation.

In this study, participants created knowledge to reduce ambiguity and gain personal growth. Participants used knowledge resources, such as databases, websites, library, and documents, to form their definition, commentary, and actions and in order to create nursing knowledge. Daneshgar and Parirokh declared that knowledge management and creation is necessary for individual improvement. Faculty members have requested to have an active role in knowledge creation (Daneshgar & Parirokh, 2007). An active knowledge management culture is required for a continuous learning process (Singh, 2008). In this study, participants created knowledge in order to learn, teach, and to apply it.

To construct knowledge, scientific library, virtual space, human resources (including educators, students, and clinical coworkers), and clinical environments were used as facilitators. Interactions and discussions in postgraduate classes provided the context for knowledge creation. Experiences of students showed that knowledge creation was the result of assignments, which prepared a base for individual growth. Nevertheless, sometimes, in post-graduation programs, research was determined as student assignment which had to be performed in a limited time period, and thus, did not result in deep learning.

The majority of organizational knowledge was reserved in people. Academic educators were considered as a source of knowledge for bachelor students. Inexperienced educators profit from experienced colleagues inside and outside the faculty as a source of knowledge. According to Walsh and Ungson, personnel information is based on direct experiences and observations and when they are recalled, they appear as clear/eloquent experiences (Chung-Hung, Dauw-Song, Bruce Chien-Ta, & Desheng Dash, 2010). Clinical environment, as a source of knowledge, provides learning opportunity, which changes perspectives.

Participants acquired and stored knowledge in their minds, and reflected on issues to build more advanced knowledge. Knowledge construction was challenged by cultural barriers, and lack of facilities and time. Culture of knowledge management could be based on the language; knowledge, ideology, and organizational attitude that determine the environment in which the information is exchanged (Nonaka, 1994). Due to the high volume of teaching courses, faculty members had less opportunity to perform research. Low speed of internet and lack of strong and updated nursing books were other barriers for knowledge creation. In a study by Walsh and Ungson, there was a significant relationship among organizational structure, human resources, organizational culture, and information technology and knowledge management (Walsh & Ungson, 1991).

Dynamics of mind and social factors are the underlying factors of knowledge creation. Dynamism of mind is determined by being critical and creative. This characteristic causes the mind to review various issues with an open mind, and therefore, to create new knowledge. The nursing practice is considered as a process concerning creative activities which construct explicit knowledge from tacit knowledge (Gharehbigloa, Shadidizajib, Yazdanic, & Khandehzamin, 2012). Based on data analysis, a positive comorbidity existed among educators, peers, and students in knowledge construction. When students had questions, they debated with their peers or educators, studied it with an open mind, and examined the answers from different perspectives in order to create new knowledge. Nonaka, Byosiere, Borucki, and Noboru recognized four different models of interaction among tacit and explicit knowledge in which existent knowledge can be converted into new knowledge (Yuqin, Guijun, Zhenqiang & Quanke, 2012; Nonaka, Byosiere, Borucki, & Noboru, 1994). Converting knowledge is presented in four styles of socialization, externalization, combination, and internalization (Pässilä, Uotila, & Melkas, 2013). Yuqin revealed that tacit knowledge is non-structured knowledge and hard to codify, it can be transferred and shared, and construct synergy (Yuqin et al., 2012).

From constructional perspective, knowledge is the co-creation of facts rather than generally accepted beliefs (Gergen, 2009). Knowledge construction has been adopted based on a social constructionist perspective (Nonaka & Krogh, 2009). In converted knowledge, socialization of any relationship between individuals is a social process for sharing knowledge and learning from each other (Nakamori, 2012). Individuals convert explicit knowledge to tacit knowledge through internalization, and in externalization, convert tacit knowledge to explicit Knowledge (Nonaka & Krogh, 2009). Through the reconfiguring of existing knowledge new knowledge can be gained; this is termed combination (Nonaka et al., 1994).

Conversion of knowledge was performed through processes of knowledge creation in the mind, reflection, research, and praxis. Participants put pieces of knowledge together and created new knowledge in their mind. Students reflected on subjects, reviewed them critically, and create new knowledge. Participants used two forms of reflection: personal reflection and contemplation, and group reflection and discussion. Reflection tends to clarify ambiguities, and group discussion, clarifies issues from different point of views. Reflection had significant effect on post-test scores (Sullivan-Marx, 2006).

Praxis involves constantly noting what occurs in practice, querying critical questions in respect to that practice, constructing change to revise practice in a desired direction, and noting what occurs as outcome (Chinn & Kramer, 2004). Moss, Grealish, and Lake have confirmed that recent graduate pedagogies change the nursing education further than strategies that look for integration of theory and practice, and create dialectic between theory and practice (Moss, Grealish, & Lake, 2010).

In praxis, practice must be cognizant of theory, and theory, in turn, cognizant of practice. Praxis is cognizant of knowledge in action. Theory that informs or leads to practice is essential in nursing. Situational theory is a theory that informs or guides practice. This theory is necessary in nursing. It means that through situational theory we can describe, explain, predict, and prescribe how nursing practice may be clarified. In doing so, it will recognize the problems that are present in nursing (McKenna & Slevin, 2008). Development of this theory explains the various elements that create professional nursing practice in order to clarify the relationship between the distinct components, and to forecast nursing practice through examination of known variables (Kalofissudis, 2007).

The last strategy for knowledge creation was research. Research was conducted by faculty members or students through dissertation. Research is the main method for knowledge creation in postgraduate courses in academia. In all stages of research, participants engage in gaining knowledge and in the end of the process knowledge is created. New knowledge can be the result of research (Ken, Christopher, & Kevin, 2005). In our study, MSc students created knowledge through quantitative research and PhD students created knowledge through qualitative and mixed methods research. Oddi, Whitley, and Pool (1994) proposed that graduation of nursing students has an essential impact on the improvement and distribution of nursing knowledge (Oddi, Whitley, & Pool, 1994). This finding is in line with the findings of the present research.

However, some research titles which are situational may lead to theory inform of practice such as the definition of the organ donation nursing given through concept synthesis and grounded theory research approach. Despite these attempts, there is gap between theory and practice. It is necessary that substantive theory be improved in order to offer a suitable basis to advance nursing practice. Substantive theory can guide the nursing practice toward a novel practice method (Chinn & Kramer, 2004).

Based on the participants’ experience, the outcome of main mental process in creating knowledge was gaining new knowledge through disconformity in order to gain more individual advantages.

Like other research studies, we also met some limitations. Some of the participants may not remember their previous experiences or be unable to tell their whole story. Hence, these findings cannot be extended to all educational nursing settings as the process of knowledge creation.

## 5. Conclusion

It is recognized that nursing knowledge creation occurred through converting knowledge in nursing educational settings. Systematic knowledge management was established in nursing education in a ways that it was considered in the mission and strategic plan of educational nursing and knowledge creation in the operational plan phase.

Regarding professional challenges, it is essential to construct applicable knowledge according to professional needs and problems. Use of systematic knowledge management and knowledge creation in nursing education will result in better teaching and learning, decision making, and problem solving in theoretical and clinical situations. Suitable situations for knowledge creation should be established through discussion, praxis, reflection, and research. Organized research is established and knowledge created by researchers in order to achieve common and individual advantage. Researches conducted by researchers must use substantive theory or situation-producing theory to improve nursing education and solve professional problems.
